# Prepartum body conditions affect insulin signaling pathways in postpartum adipose tissues in transition dairy cows

**DOI:** 10.1186/s40104-019-0347-4

**Published:** 2019-05-15

**Authors:** Fanjian Zhang, Dan Li, Qiong Wu, Jian Sun, Wenyi Guan, Yinxu Hou, Yaohong Zhu, Jiufeng Wang

**Affiliations:** 1 Beijing Vocational College of Agriculture, Beijing, 102442 People’s Republic of China; 2 College of Veterinary Medicine, China Agricultural University, Beijing, 100193 People’s Republic of China; 3 Animal Science and Technology College, Beijing University of Agriculture, Beijing, 102206 People’s Republic of China

**Keywords:** Adipose tissue, Body condition score, Insulin signaling pathway, Transition dairy cow

## Abstract

**Background:**

Overconditioned dairy cows are susceptible to excessive lipolysis and increased insulin resistance during the transition period. The associations among body fat reserve, insulin resistance, and lipolysis in adipose tissues (AT) remain to be elucidated. Therefore, this study aimed to investigate whether excessive fat reserves influence the insulin signaling pathway in AT postpartum.

**Results:**

Twenty multiparous dairy cows were selected and assigned to one of two groups, according to prepartum body condition score (BCS): Control group (BCS = 3.0–3.5; *n* = 10) and Overconditioned group (BCS ≥ 4.0; *n* = 10). Blood samples were collected on days −14, −7, −4, −2, −1, 0, 1, 2, 4, 7, and 14 relative to parturition. Subcutaneous AT were collected on day 2 following parturition for quantitative real-time polymerase chain reaction and western blot analyses. No differences were observed between the two groups in serum glucose, non-esterified fatty acids, β-hydroxybutyric acid, tumor necrosis factor (TNF) α, insulin, or leptin concentrations during the experimental period. Compared with the control cows, the overconditioned cows had lower serum triglyceride levels and higher adiponectin concentrations. In the AT postpartum, insulin receptor mRNA and protein levels were lower in the overconditioned cows than in the control cows, and no differences were found in glucose transporter 4 mRNA. Compared with the control cows, the overconditioned cows had lower mRNA levels of TNFα and higher mRNA levels of peroxisome proliferator-activated receptor gamma (PPARγ) in AT postpartum. The phosphorylated protein kinase B (AKT) content and phosphorylation rate of AKT were increased in the overconditioned cows compared with the control cows, which suggested that the downstream insulin signaling in AT was affected.

**Conclusions:**

In the present study, transition dairy cows with higher BCS did not show more fat mobilization. The changes of insulin signaling pathway in AT postpartum of overconditioned cows may be partly related to the expression of PPARγ and TNFα, and the secretion of adiponectin.

## Background

During transition from late pregnancy to early lactation, most dairy cows, especially high-yielding dairy cows which need more energy for milk production, undergo negative energy balance (NEB), leading to the mobilization of fatty acids from adipose tissues (AT) [1]. AT are important for the dynamic control of energy metabolism, and adequately regulated lipolysis is necessary for dairy cows to successfully adapt to NEB, and the limited release of non-esterified fatty acid (NEFA) can fully meet the energy demand [2, 3]. However, excessive lipolysis can lead to accumulation of high concentrations of NEFA and beta hydroxybutyric acid (BHBA) in the blood, which can result in health problems such as ketosis and fatty liver [4, 5] and potential losses in milk yield [6, 7].

Insulin resistance (IR) can accelerate AT lipolysis and the accumulation of NEFA in turn increases the degree of IR, which is associated with the development of inflammatory and metabolic diseases [8]. Transition dairy cows with an excessive lipolytic response exhibit impaired insulin signaling in AT [9]. Therefore, maintaining a balance between IR and fat mobilization is beneficial to the metabolic adaptation of transition dairy cows. The regulation of insulin signaling pathway by blood metabolites during lipolysis may be responsible for the development of IR in AT [10, 11].

The body condition score (BCS) is recognized as an important variable in transition dairy cow management [12], and a higher BCS, namely obesity, indicates a greater risk for postpartum metabolic disorders [13–15]. The influence of nutrition factors on insulin sensitivity [16] and insulin signaling [10] has been extensively investigated in dairy cows. Overconditioned cows are known to be susceptible to excessive fat mobilization during transition period [17]. Although previous studies have suggested that overfed cows or cows with a higher BCS are more insulin resistant [18, 19], it was reported recently that body condition did not affect AT IR postpartum [11]. Studies have also suggested that cows with high weight loss were more prone to specific IR in AT [9]. AT also play endocrine roles by secreting adipokines, including adiponectin, leptin and tumor necrosis factor (TNF) α, which are involved in the regulation of AT metabolism [20]. Peroxisome proliferator-activated receptor gamma (PPARγ), a subtype of the PPAR family, is highly expressed in ruminant AT and has been shown to play a central role in the transcriptional control of genes encoding proteins involved in glucose and lipid metabolism [21–23]. The metabolic changes of transition dairy cows with different BCS may be related to IR development.

Considering that the interaction mechanism of IR and lipolysis is still unclear, we hypothesized that excessive lipolysis happened in overconditioned cow, and their blood metabolites affected the development of IR in AT postpartum, especially during the first wk after calving because of sudden increase in energy demand at the initiation of milk synthesis [24]. The objective of the present study was to investigate the association of a higher BCS prepartum with dynamic changes in metabolites, hormones, and adipokines in transition dairy cows, and to reveal whether excessive fat reserves influence the insulin signaling pathway at the gene and protein levels in AT immediate postpartum.

## Materials and methods

### Animals and diets

This study was carried out in a high-yielding commercial dairy herd in Beijing, China. Prepartum BCS was assessed according to a 5-point scale method [23] on approximately day −21 relative to the expected calving date. A total of 20 clinically healthy, multiparous (parity from 2 to 4) Holstein cows, with no history of debilitating disease, were selected and assigned to one of two groups according to the scored BCS as follows: control group (BCS = 3.0–3.5; *n* = 10) and overconditioned group (BCS ≥ 4.0; *n* = 10). All the selected cows had free access to water throughout the study and were fed three times daily (07:00, 12:00 and 19:00 h) with a total mixed ration ad libitum. Table 1 reports the composition of the diet used during the experiment (close-up dry period, 20d prior to expected parturition; lactation period, days following parturition). During the lactation period, the cows were milked at 05:30, 10:30, and 17:00 h. Milk yield for each experimental cow was recorded daily from day 11 to day 30 relative to parturition.

**Table 1:** Ingredient and nutrient composition (DM basis) of the basal diet for dairy cows during the experimental period

Item^a^	Close-up dry period	Lactation period
Ingredient, %		
Roughage	56.8	45.0
Concentrate	43.2	55.0
Chemical analysis		
NE_L_, Mcal/kg	1.47	1.76
DM, %	58.7	53.8
CP, %	14.2	17.4
Fat, %	3.1	6.1
NDF, %	52.9	33.4
ADF, %	28.1	19.6
Calcium, %	0.42	0.8
Phosphorus, %	0.44	0.3

^a^*DM* Dry matter, *NEL* Net energy for lactation, *CP* Crude protein, *NDF* Neutral detergent fiber, *ADF* Acid detergent fiber

### Blood and adipose tissue sampling

Blood samples were collected from the caudal vein prior to the morning feeding on days −14, −7, −4, −2, −1, 0, 1, 2, 4, 7, and 14 relative to parturition. The samples were centrifuged (2,000×*g* for 10 min) and the serum was collected and stored at −20 °C until analysis.

AT samples of approximately to 5 g were collected on day 2 postpartum from the area below the spinal processes between the ischium (pin bone) and coccygeal vertebrae of the three cows in each group, as previously described [25]. The AT samples were immediately placed into plastic vials, snap-frozen in liquid nitrogen and stored at −80 °C.

### Serum metabolite, hormone, and adipokine analysis

The concentrations of glucose and triglycerides were determined for each serum sample using commercially available kits (Biosino Bio-technology and Science Inc., Beijing, China) in an automatic clinical chemistry analyzer (Accute TBA-40FR, Toshiba, Tokyo, Japan).

Serum concentrations of NEFA (Cusabio, Wuhan, China), BHBA (Cusabio, Wuhan, China), TNF-α (Abcam, Cambridge, UK), insulin (Colorfulgene Biological Technology, Ltd., Wuhan, China), leptin (Colorfulgene Biological Technology, Ltd., Wuhan, China), growth hormone (GH; Cusabio, Wuhan, China), and adiponectin (Colorfulgene Biological Technology, Ltd., Wuhan, China) were determined using commercially available ELISA kits, according to the manufacturer’s instructions.

### RNA extraction and reverse transcription

Total RNA was extracted from the AT samples using TRIzol reagent (Invitrogen, Carlsbad, CA, USA), following the manufacturer’s instructions. The concentration and purity of the total RNA were determined spectrophotometrically at 260/280 nm. The RNA purity was assessed by its A260/A280 ratio with expected values between 1.8 and 2.0 using a NanoDrop ND-2000C spectrophotometer (NanoDrop Technologies Inc., Wilmington, DE, USA). The RNA integrity was measured using agarose gel electrophoresis and the gel image showed the distinct intact bands of 5S, 18S and 28S rRNA. The total RNA was reversed transcribed into cDNA using the ImProm-II Reverse Transcription kit (Promega, Madison, WI, USA), according to the manufacturer’s instructions. The synthesized cDNA was stored at −20 °C prior to real-time polymerase chain reaction (PCR) analysis.

### Quantitative real-time PCR analysis

Quantitative real-time PCR analysis was performed using an ABI 7500 Real-Time PCR system (Applied Biosystems, Foster City, CA, USA). Table 2 lists the sequences of primers used in this study. The cDNA was amplified with SYBR® Premix DimerEraser™ (Takara Biotechnology, Inc., Shiga, Japan) containing 2 μL cDNA, 1.0 μmol/L primers, 10 μL 2× SYBR Premix DimerEraser, and 0.4 μL ROX (passive reference dye). The templates were amplified following preincubation at 95 °C for 30 s, followed by amplification for 39 cycles (95 °C for 5 s, 60 °C for 30 s, and 72 °C for 15 s). All the reactions revealed a single product as determined by melting curve analysis. All the reactions were performed in triplicate.

**Table 2:** Sequences and accession numbers of oligonucleotide primers used for real-time PCR and the length of the PCR products

Gene name	Oligonucleotide sequences (5’to3’) of primers	GenBank accession number	Product length, bp
*GAPDH*	F: CCACGTTGTAGCCGACATC	NM001034034	201
	R: CCCTGAAGAGGACCTGTGAG		
*β-actin*	F: CACCGCAAATGCTTCTAGGC	NM_173979.3	186
	R: TGTCACCTTCACCGTTCCAG		
*HPRT*	F: GACCAGTCAACAGGCGACAT	NM_001034035.2	130
	R: TGACCAAGGCAAGCAAAGTC		
*INSR*	F: AGGAGCTGGAGGAGTCCTCGTTCA	XM005208817.2	147
	R: CATTCCCCACGTCACCAAGGGCTC		
*GLUT4*	F: TTCATTGGCGCCTACTCAGG	NM174604.1	142
	R: CTAGCACCTGGGCGATTAGG		
*TNFα*	F: CCACGTTGTAGCCGACATC	NM173966	155
	R: CCCTGAAGAGGACCTGTGAG		
*PPARγ*	F: ACTTTGGGATCAGCTCCGTG	NM181024.2	137
	R: GTCAGCTCTTGGGAACGGAA		

*GAPDH* Glyceraldehydes 3-phosphate dehydrogenase, *HPRT* Hypoxanthine phosphoribosyl-transferase, *INSR* insulin receptor, *GLUT4* Glucose transporter 4, *TNFα* Tumor necrosis factor-alpha, *PPARγ* Peroxisome proliferator-activated receptor gamma

The relative abundance of mRNA was calculated according to the method of Li et al. [26]. Glyceraldehyde-3-phosphate dehydrogenase (*GAPDH*), β-actin and hypoxanthine phosphoribosyl-transferase (*HPRT*) were chosen as the housekeeping control genes. To evaluate the relative quantification of mRNA expression, the cycle threshold (C_T_) values of the target genes were normalized to the geometric mean of the C_T_ values of the three selected housekeeping genes, and the results were presented as fold changes using the 2^−ΔΔCT^ method. The relative mRNA expression of the target genes in each group was calculated using the following equations:

$$ {\Delta \mathrm{C}}_{\mathrm{T}}={\mathrm{C}}_{\mathrm{T}\left(\mathrm{target}\ \mathrm{gene}\right)}-{\mathrm{C}}_{\mathrm{T}\left(\mathrm{geometric}\ \mathrm{mean}\ \mathrm{of}\ \mathrm{housekeeping}\ \mathrm{gene}\mathrm{s}\right)},{\Delta \Delta \mathrm{C}}_{\mathrm{T}}={\Delta \mathrm{C}}_{\mathrm{T}\ \left(\mathrm{treated}\ \mathrm{group}\right)}-{\Delta \mathrm{C}}_{\mathrm{T}\ \left(\mathrm{control}\ \mathrm{group}\right)}. $$

### Western blotting

Proteins were extracted from the AT using a commercial kit for AT (Invent Biotechnologies, Inc., Plymouth, MN, USA), according to the manufacturer’s instructions. A BCA protein assay kit (Pierce Chemical Co., Rockford, IL, USA) was used to determine the concentration of protein in the supernatant from each sample. The following primary antibodies were used: rabbit polyclonal anti-protein kinase B (AKT) (#9272S; 1:1,000 dilution), rabbit monoclonal anti-phospho(p)-AKT (Ser473) (#9272; 1:2,000 dilution), rabbit monoclonal anti-p-AKT (Thr308) (D25E6; 1:1,000 dilution), mouse monoclonal anti-INSR (ab69508; 1:1,000 dilution), rabbit polyclonal anti-insulin receptor substrate 1 (IRS1) (#2382; 1:1,000 dilution), rabbit polyclonal anti-p-IRS1 (#2381; 1:1,000 dilution) (Cell Signaling Technology, Inc., Danvers, MA, USA), and mouse anti-β-actin Mab (66009–1-Ig; 1:1,000 dilution; Proteintech Group Inc., Chicago, IL, USA). Horseradish peroxidase-conjugated goat anti-mouse IgG(H + L) (SA00001–1; 1:5,000 dilution) or goat anti-rabbit IgG(H + L) (SA00001–2; 1:5,000 dilution) (Proteintech Group, Inc.) were used as secondary antibodies. The intensity of the bands was quantified by densitometry analysis using Quantity One software (Bio-Rad Laboratories, Inc., Hercules, CA, USA). The results are presented as the ratio of the INSR band intensity to the β-actin band intensity, and the ratio of the p-IRS1, p-AKT (Thr308) or p-AKT (Ser473) band intensity to IRS1 or AKT band intensity, respectively.

### Statistical analysis

The PROC MIXED procedure of SAS (SAS Institute, Inc., Cary, NC, USA) was used to analyze data for serum variables. The statistical model included day (day relative to parturition, D), BCS (control and overconditioned, B), and the interaction of BCS and day (B × D) as fixed effects, and cows within the BCS group as a random effect. The GLM procedure of SAS was used to analyze data for gene and protein expression levels in AT. Student’s *t* test was used to compare differences between the least square means. *P* < 0.05 was considered statistically significant.

## Results

The BCS was statistically different between the control (3.2 ± 0.1) and overconditioned (4.2 ± 0.2) groups (*P* < 0.01). The average daily milk yield of overconditioned cows was lower than control cows (38.4 ± 0.7 and 41.9 ± 0.6 kg/d respectively, *P* < 0.01).

### Serum glucose, triglycerides, NEFA, BHBA, and TNFα

**Fig. 1:**
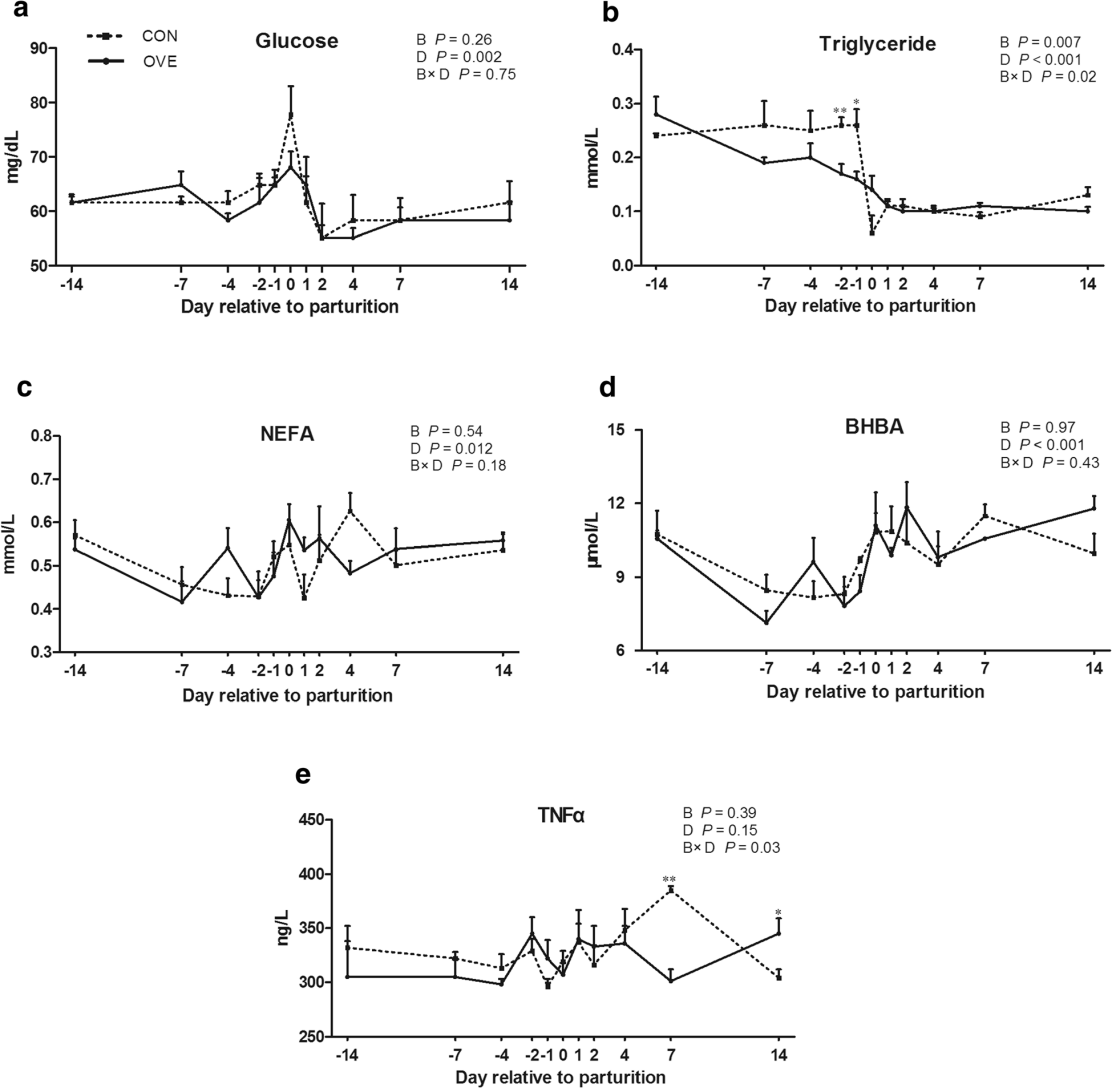
Effects of prepartum BCS on serum glucose (**a**), triglycerides (**b**), NEFAs (**c**), BHBA (**d**), and TNFα (**e**) in transition dairy cows. Error bars indicate the standard error of the mean. Significant difference between CON (BCS = 3.0–3.5) and OVE (BCS ≥ 4.0) groups on the same sampling day are noted with * (B × D *P* < 0.05) or ** (B × D *P* < 0.01); B, BCS; D, day (day relative to parturition); B × D, interaction between BCS and day relative to parturition; CON, control; OVE, overconditioned; NEFA, non-esterified fatty acid; BHBA, beta hydroxybutyric acid; TNF-α, tumor necrosis factor α

The BCS had no significant effect on serum glucose concentration (Fig. 1a). Serum glucose concentration increased gradually from day −4 and was highest on day 0 (*P* < 0.05), then decreased until day 4. The serum triglycerides concentration was lower in the overconditioned cows (*P* = 0.007) than in the control cows, particularly due to differences during the prepartum period. There was a B × D interaction (*P* = 0.02) in the triglycerides concentration due to a gradual decrease from day −14 to day −1 in the overconditioned cows (Fig. 1b). Serum NEFA and BHBA concentrations during the postpartum period were higher (*P* < 0.05) than those during the prepartum period. No differences were found between the two groups in serum NEFA and BHBA concentrations (Fig. 1c and d). There were no overall BCS or day effect on serum TNFα concentration (Fig. 1e). A B × D interaction effect (*P* = 0.03) was observed for the TNFα concentration due to a decrease on day 7 in the overconditioned cows compared with an increase for the control cows.

### Serum hormone and adipokine concentration

**Fig. 2:**
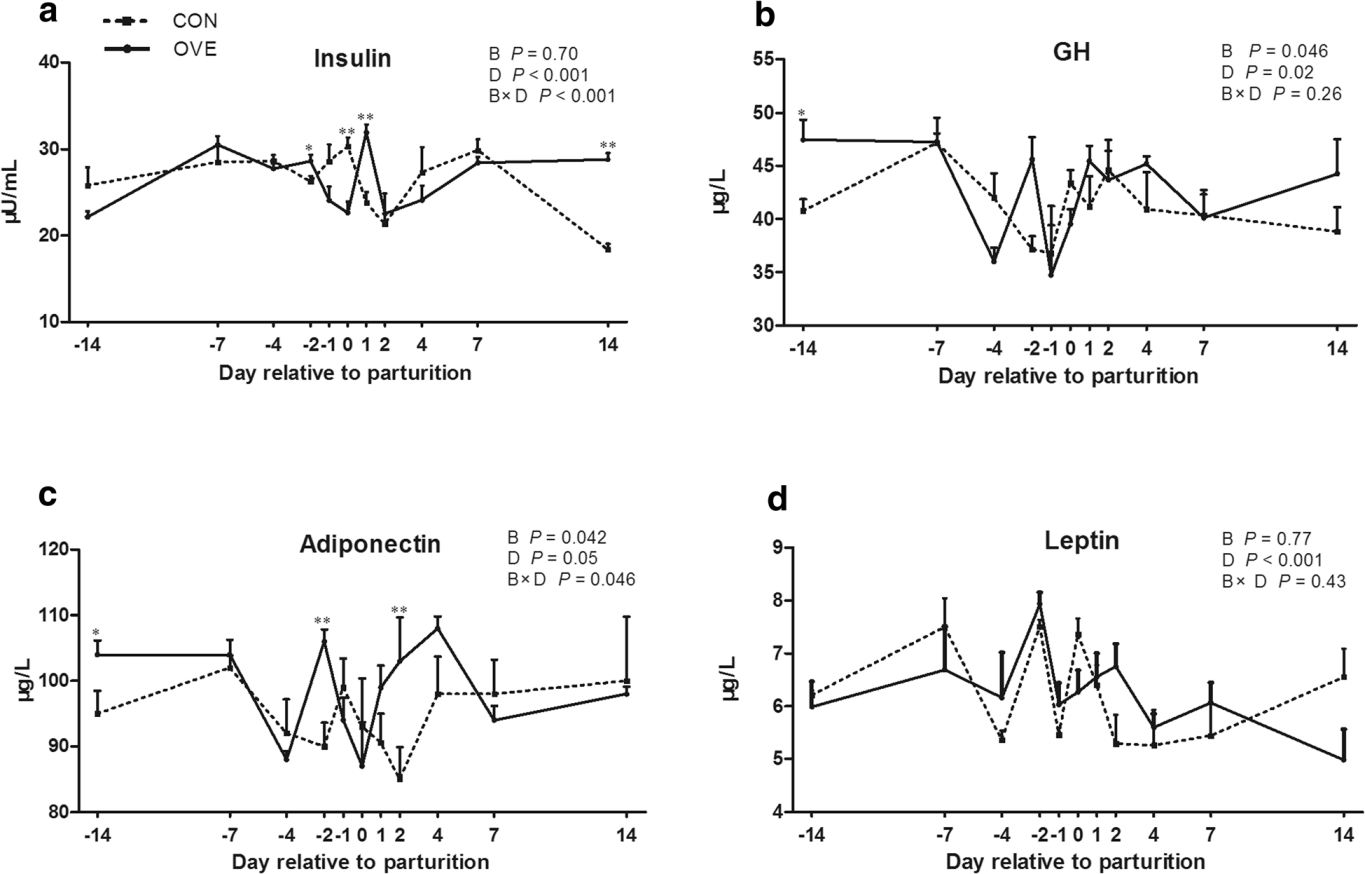
Effects of prepartum BCS on serum insulin (**a**), GH (**b**), adiponectin (**c**), and leptin (**d**) in transition dairy cows. Error bars indicate the standard error of the mean. Significant difference between CON and OVE groups on the same sampling day are noted with * (B × D *P* < 0.05) or ** (B × D *P* < 0.01). GH, growth hormone

Serum insulin concentrations in the control and overconditioned groups decreased to a lower level on day 2 (*P* < 0.01) (Fig. 2a). There was a significant B × D interaction (*P* < 0.001) in the serum insulin level due to a gradual decrease in the overconditioned group from day − 2 to day 0. On day 14, the insulin concentration in the overconditioned cows was higher (*P* < 0.01) than that in the control cows.

The serum concentrations of GH and adiponectin in the overconditioned cows were higher overall than those in the control cows during the experimental period (*P* = 0.046 and 0.042, respectively) (Fig. 2b and c). The GH concentrations during the postpartum period were higher (*P* < 0.05) than that on day −1. For the adiponectin concentration, the overconditioned cows showed a gradual increase from day 0 to day 4 compared with a decrease in the control cows, which accounted for the B × D interaction effect (*P* = 0.046) for serum adiponectin concentration.

Serum leptin concentrations in the control and overconditioned cows gradually decreased (*P* < 0.05) following parturition. No significant difference in leptin concentrations was found between the control and overconditioned groups (Fig. 2d).

### Gene and protein expression in adipose tissue

**Fig. 3:**
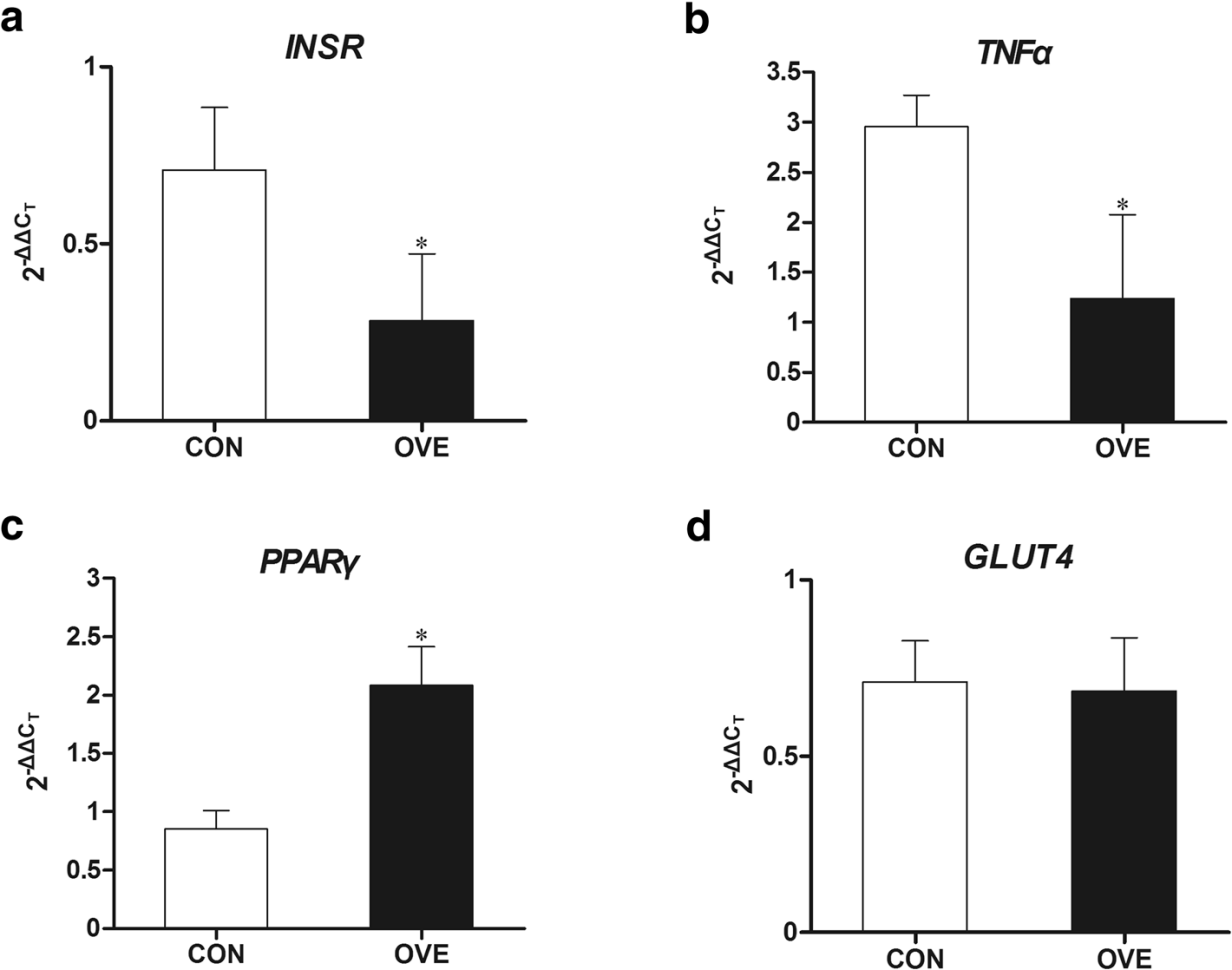
Quantitative real-time PCR analysis of *INSR* (**a**), *TNFα* (**b**), *PPARγ* (**c**), and *GLUT4* (**d**) in subcutaneous adipose tissues. The tissues were collected on day 2 postpartum from CON (*n* = 3) and OVE (*n* = 3) cows. Error bars indicate the standard error of the mean. Significant differences between CON and OVE groups are noted with *(*P* < 0.05). INSR, insulin receptor; PPAR, peroxisome proliferator-activated receptor; GLUT4, glucose transporter 4

Compared with the control cows, the relative mRNA expression levels of insulin receptor (*INSR*) and *TNFα* were decreased (*P* = 0.046 and *P* = 0.04 respectively, Fig. 3a and b), and the mRNA expression of *PPARγ* was increased (*P* = 0.03, Fig. 3c) in the AT from the overconditioned cows postpartum. No differences were found in the relative mRNA expression of glucose transporter 4 (*GLUT4*) between the two groups (Fig. 3d).

**Fig. 4:**
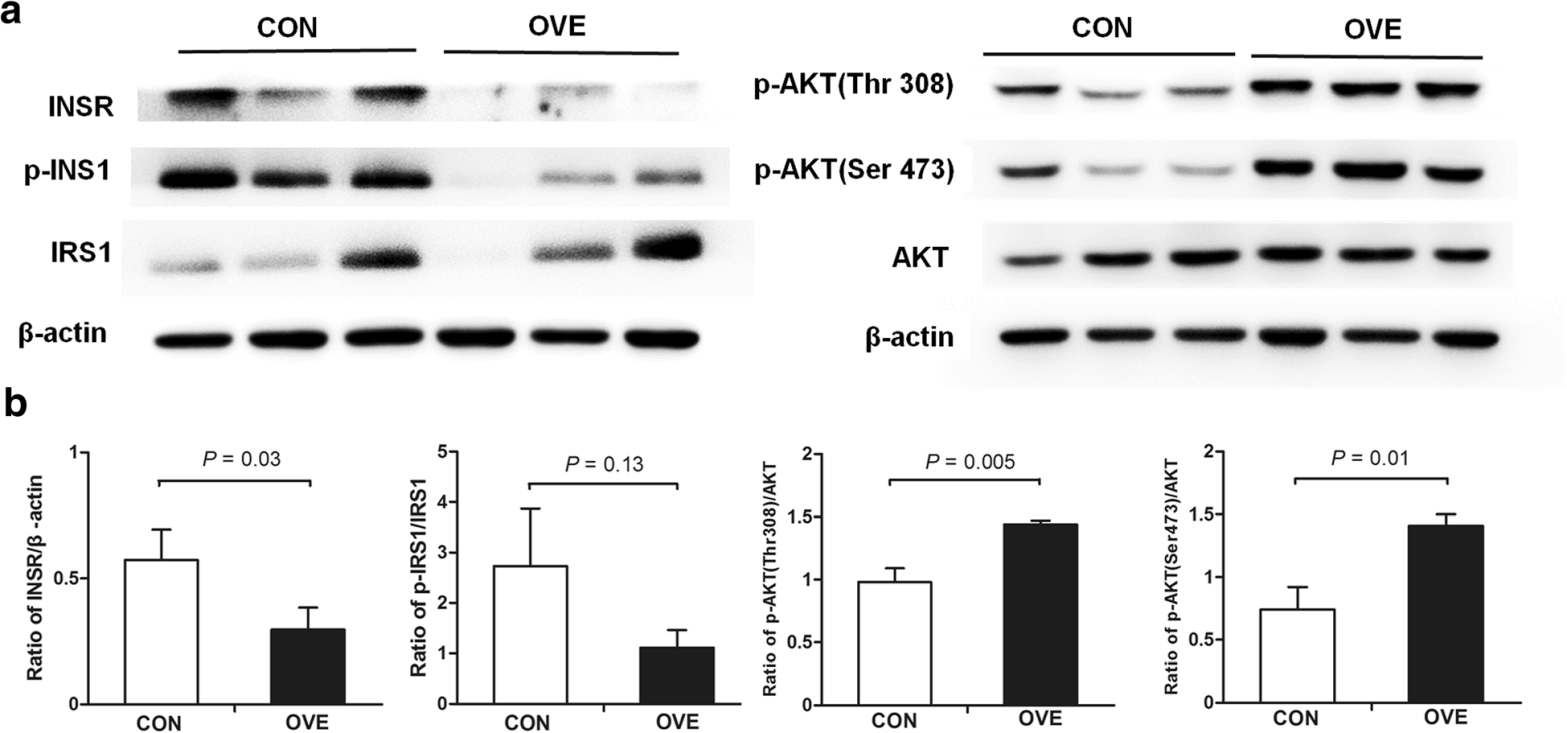
Western blot detection of insulin signaling proteins in subcutaneous adipose tissues. These tissues were collected on day 2 postpartum from CON cows (*n* = 3) and OVE cows (*n* = 3). **a** Panels of INSR, IRS1, p-IRS1, AKT, p-AKT (Thr308) and p-AKT (Ser473) protein. β-actin was measured as an internal control. **b** Intensities of INSR, IRS1, p-IRS1, AKT, p-AKT (Thr308) and p-AKT (Ser473) bands were determined using Quantity One software. The results are presented as the ratio of INSR band intensity to the β-actin band intensity, the ratio of p-ISR1 band intensity to IRS1 band intensity, and the ratio of p-AKT (Thr308) and p-AKT (Ser473) band intensities to the AKT band intensity. IRS1, insulin receptor substrate 1; AKT, protein kinase B

The postpartum AT from the overconditioned cows had lower protein expression of INSR than those from control cows (*P* = 0.03, Fig. 4a and b). The higher ratios of p-AKT (Thr308):AKT (*P* = 0.005) and p-AKT (Ser473):AKT (*P* = 0.01) were observed in the postpartum AT from the overconditioned cows. No significant difference (*P* = 0.13) was found in the p-IRS1:IRS1 ratio between the two groups.

## Discussion

Elevated blood NEFA concentration is one important indicator of NEB related to adipose mobilization [5, 27]. Consistent with previous studies [28, 29], postpartum dairy cows in this study have been in a state of NEB and high lipolytic rate, as indicated by elevated serum NEFA and BHBA concentration. Contrary to initial expectations, overconditioned cows did not show higher serum NEFA than control cows, which is not consistent with previous studies [13, 15, 30]. Overconditioned cows in present study showed lower milk yield, which could partly indicate a lower NEB state. Unfortunately, we did not continuously measure milk production and dry matter intake throughout the experimental postpartum period, which are useful for calculating accurately the degree of NEB [5]. Additionally, lipolysis can be divided into basal and demand lipolysis, and demand lipolysis is the main source of blood NEFA in transition dairy cows, which is mainly modulated by hormone sensitive lipase pathway [2]. The assessment of hormone sensitive lipase activity in AT could better reflect the level of lipolysis, which should be considered for future study. It has been suggested that the predisposition for intense lipolytic responses has a genetic basis [31]. Previous study showed that BCS loss, rather than BCS, was positivity associated with an intense lipolysis rate during the transition period [9, 32]. Unfortunately, the changes of BCS in our study were not measured during the experimental period. Future study is required to evaluate the association among the rate of BCS change, lipolysis and insulin signaling pathway throughout the transition period. With the approach of calving, overconditioned cows exhibited gradual decrease in serum triglycerides levels. It has been reported that fatty liver in transition cows is associated with decreased plasma triglycerides concentration [33, 34]. These results indicated that prepartum overconditioned cows were at a higher risk of developing fatty liver, as suggested by Drackley [35].

In our study, overconditioned cows had higher serum adiponectin concentrations than the control cows, consistent with the results of previous studies showing increased expression of adiponectin in the AT of cows with a higher BCS [36] or overfed cows with high BHBA concentrations [10] during the peripartum period. Adiponectin, a type of adipokine secreted exclusively from AT and abundant in plasma [37], is recognized as an insulin-sensitizing hormone, improving whole-body insulin sensitivity in models of genetic and diet-induced obesity via the activation of AMP protein kinase signaling [38]. In human studies, decreased adiponectin levels in the plasma or AT have been observed in obese individuals [39] and patients with type 2 diabetes [40], which shows an association between adiponectin levels and obesity-related metabolic dysfunction. A positive association has also been found between serum adiponectin levels and insulin responsiveness to glucose and fatty acid in dairy cows during the dry period [41]. In the same study, the BCS and serum adiponectin concentration showed a negative correlation [41], which was in agreement with a recent study, showing higher adiponectin gene expression in cows with a lower BCS [30]. During postpartum period in present study, although the rate of lipolysis increased in both groups, the changes of adiponectin were different, that is, the concentration of adiponectin increased in overconditioned cow, but decreased in control cow. It is still unclear whether the improvement of metabolic function observed in the overconditioned cows is due to the protection of adiponectin, or the lower level of NEB and lower milk production. Further *in vivo* and *in vitro* studies should be considered to reveal whether and how adiponectin improve metabolic function in transition cows.

PPAR-γ, a central regulator of adipocyte biology and energy homeostasis, can induce adipocyte differentiation by activating the expression of adipocyte-specific genes and is also known as an insulin sensitizer [10]. The administration of thiazolidinediones, which are synthetic PPARγ ligands, significantly increased plasma adiponectin concentrations in insulin-resistant humans and rodents [42]. Previous studies on dairy cows [22, 43] and dairy steers [44] have confirmed the insulin sensitivity effect of PPARγ. Postpartum overconditioned cows in the present study showed higher expression of PPARγ in AT, which is in accordance with previous reports [23, 36]. We assumed that the higher serum adiponectin concentrations mentioned above were regulated by the elevated gene expression of PPARγ in the AT of overconditioned cows. In contrast, a recent study has indicated that the expression of PPARγ in AT did not differ significantly between the cows fed a high-energy diet and those fed a controlled-energy diet [10], which was consistent with the results from a study by Selim et al. [45]. The regulatory mechanism of PPARγ under different nutritional conditions remains to be fully elucidated.

Studies in humans have suggested that adipose-derived TNFα represents a link among obesity, inflammation, and diabetes, and increased expression levels of TNFα in AT of obese subjects have been strongly implicated in the pathogenesis of IR [46, 47]. The increase in the expression of TNFα in AT has been found to be inhibited by PPAR agonists, *in vitro*, suggesting that the expression of TNFα is regulated by the activation of PPAR [48]. In the present study, overconditioned cows showed lower TNFα not only at the gene expression level in AT but also in serum during the immediate postpartum period. Adiponectin and TNFα may antagonize each other or perform opposite functions locally in AT, as suggested by Maeda et al. [42].

Insulin is the most potent anabolic hormone, and promotes the synthesis and storage of carbohydrates, lipids, and proteins, while inhibiting their degradation and release into the circulation. In AT, insulin signal transduction starts with binding insulin to INSR. The consequent intracellular cascade, including the phosphorylation of IRS1, interaction with phosphatidylinositol 3-kinase, and the activation of AKT by phosphorylation at Thr308 and Ser473, promotes the expression and translocation of insulin-dependent GLUT4, responsible for insulin-induced glucose uptake from the blood in AT [49]. Insulin resistance can be assessed by insulin responsiveness, which can be evaluated at the receptor level, and insulin sensitivity, which can be evaluated at the post-receptor level [50]. In the AT postpartum, insulin receptor mRNA and protein levels were lower in the overconditioned cows, indicating that insulin response to glucose might decrease. Lower mRNA levels of INSR in AT were also found in the overfed cows on day 21 postpartum when compared with the normal fed cows [51]. Interestingly, the overconditioned cows had increased p-AKT content and an increased phosphorylation rate of AKT in the AT which indicated that AT responsiveness to insulin was likely to increase. A previous study involving 3 T3-L1 adipocytes demonstrated that the suppression of PPARγ reduced insulin-stimulated glucose uptake by affecting the downstream activation of AKT, without affecting the early insulin signaling steps in the adipocytes [52]. It is unclear whether PPARγ play regulation role in maintaining a balance between IR and fat mobilization in transition dairy cows. *In vitro* study is required to further reveal the association among PPARγ, lipolysis and insulin resistance in AT of transition dairy cows. Furthermore, different AT depots of cows may differentially influence the regulation of insulin sensitivity during lactation, and gene expression of adiponectin receptor 1 and TNFα were mostly different in retroperitoneal AT [53]. Different adaptations of cows during the transition period based on different AT depots, even of different subcutaneous AT origin should be considered for further studies.

## Conclusion

No differences in serum NEFA and BHBA concentrations were observed between the overconditioned and control cows during transition period. The concentration of serum adiponectin was higher in the overconditioned cows than in the control cows. In the AT postpartum, the overconditioned cows showed lower gene and protein expression levels of INSR and no differences were found in GLUT4 gene expression. The p-AKT content and ratios of p-AKT:AKT were increased in the overconditioned cows, suggesting acti vation of the downstream insulin signaling pathway. Meanwhile, a lower gene expression of TNFα and higher expression of PPARγ were found in AT postpartum of overconditioned cows. The changes of insulin signaling pathway in AT postpartum may be partly related to the expression of PPARγ and TNFα, and the secretion of adiponectin.

## Abbreviations

ADF: Acid detergent fiber
AKT: Protein kinase Bs
AT: Adipose tissue
BCS: Body condition score
BHBA: Beta hydroxybutyric acid
CP: Crude protein
DM: Dry matter
GAPDH: Glyceraldehyde-3-phosphate dehydrogenase
GH: Growth hormone
GLUT4: Glucose transporter 4
INSR: Insulin receptor
IR: Insulin resistance
IRS1: Insulin receptor substrate 1
NDF: Neutral detergent fiber
NEB: Negative energy balance
NEFA: Non-esterified fatty acid
NEL: Net energy for lactation
PCR: Polymerase chain reaction
PPAR: Peroxisome proliferator-activated receptor
TNFα: Tumor necrosis factor α
